# Core Gut Microbiota of Shrimp Function as a Regulator to Maintain Immune Homeostasis in Response to WSSV Infection

**DOI:** 10.1128/spectrum.02465-21

**Published:** 2022-04-12

**Authors:** Siyuan Zhang, Xumei Sun

**Affiliations:** a School of Marine Science, Ningbo University, Ningbo, People’s Republic of China; University of Georgia

**Keywords:** gut microbiota, metabolome, immune response, WSSV, shrimp

## Abstract

The gut microbiota is an integral part of the host and has a functional potential in host physiology. Numerous scientific efforts have opened new horizons in gut microbiota research and enhanced the understanding of host-microbe interactions in vertebrates. However, evidence on the association between the gut microbiota and immunity in invertebrates, especially in shrimp, which is an important aquatic animal, is limited. Herein, we conducted a comprehensive analysis based on 16S rRNA gene sequencing and liquid chromatography-coupled mass spectrometry (LC-MS) to investigate the correlation between them. Comparing the gut microbiota among the four different species of shrimp, we found huge variations and determined a core gut microbiota composed of 55 microbes. The environmental challenge of white spot syndrome virus (WSSV) infection led to changes in core microbial structures, but the alteration of core microbiota among different shrimp followed the same trend and showed immune-related function in the prediction of its metabolic potential. In metabolomic analysis, nine significantly upregulated metabolites found after viral infection indicated that they have antiviral potential. Moreover, we found a tight correlation between them and almost half of the core microbiota. These data demonstrate that these metabolites are responsible for maintaining the immune homeostasis of the host and prove the function of the gut microbiota and the related metabolome in antiviral immunity of shrimp.

**IMPORTANCE** Abundant gut microorganisms constitute a complex microecosystem with the intestinal environment of the host, which plays a critical role in the adjustment of various physiological states of the organism. Sequencing and mass spectrometry data collected from intestinal samples of shrimp after virus infection helped to investigate the special function of the microecosystem and suggested that the gut microbiota has a functional potential in maintaining immune homeostasis of the host under environmental challenge.

## INTRODUCTION

The gut microbiota is a complex microbial ecosystem with important roles in health and development of organisms (1–3). Some deterministic and stochastic processes are thought to shape the gut microbiota. These processes are generally driven by environmental and biological factors. Factors such as host immune system, pH in the gut, and dietary composition are considered to be the dominant factors in shaping the gut microbiota, processes also termed environmental selection (4, 5). Biological factors, for example, interspecies interactions (competitive, mutualistic, and some synergistic interactions) may further affect the composition of the microbiota (6–8). Under the synergistic effect of these processes, the gut microbiota in different habitats can be divided into core microbes and habitat-specific microbes (9–11). The gut microbiota profoundly regulates homeostasis mechanisms by assisting the establishment of the intestinal epithelial barrier and maintenance of immune homeostasis in hosts (12, 13). In the human body, previous studies indicated intense communication between the gut microbiota and intestinal epithelial cells and immune cells shaped specific immune responses to antigens, balancing tolerance and effector immune functions (14). For the regulation of immunity by the gut microbiota, the wide range of secondary metabolites produced by the commensal gut microbiota were proven to be crucial for host physiology and host immunity regulation (15). To date, numerous studies of the gut microbiota and its functional potential have been conducted on vertebrates, and information concerning invertebrates is limited, especially in aquatic invertebrates.

Shrimp are one of the most important animals in aquatic aquaculture (16). Although the farming industry of shrimp has increased considerably in recent years, with the expansion of farming, threats by various environmental challenges restrict the sustainable development of the industry worldwide, such as pathogen infection (17). White spot syndrome virus (WSSV) is a typical pathogen of shrimp and with a wide range of hosts (18). Infection with WSSV causes white spot syndrome of shrimp and leads to 100% mortality within 7 to 10 days (19). As reported, the gut microbiota plays a key role in regulating the immune system of the host (14). Multiple pathways such as carbohydrate metabolism and cofactor/vitamin biosynthesis that the gut microbiota participates in can promote host metabolism and anti-infection and anti-inflammation processes and regulate autoimmune reactions (20). Secondary metabolites produced by the gut microbiota include short-chain fatty acids (SCFAs), polyamines, the aryl hydrocarbon receptor (AHR), and so on (21) and can interact with host cells through the intestinal epithelia, thus influencing immune responses and disease risk (22). A few studies have found that the gut microbiota is related to growth and development in shrimp, and gut microbiota dysbiosis is responsible for shrimp white feces syndrome (16, 23, 24). Several studies have reported the impact of WSSV infection on the intestinal microbiota in Litopenaeus vannamei. However, few studies have assessed the gut microbiota associated with host immunity responses to WSSV infection.

Here, WSSV infection was used as an environmental challenge for four different species of shrimp (*Marsupenaeus japonicus*, Litopenaeus vannamei, *Macrobrachium rosenbergii*, and *Procambarus clarkii*). By using 16S rRNA gene sequencing and liquid chromatography-coupled mass spectrometry (LC-MS), the gut microbiota and metabolome before and after virus infection were identified. We determined a group of microbes that perform immunomodulatory functions in the gut of different species of shrimp and assist the host in maintaining environmental adaption. These valuable findings greatly enhanced our understanding of the functions of the gut microbiota in maintaining host fitness under environmental challenge and provide a new strategy for the prevention and treatment of viral infection in shrimp.

## RESULTS

### Gut microbiota of different shrimp.

**FIG 1:**
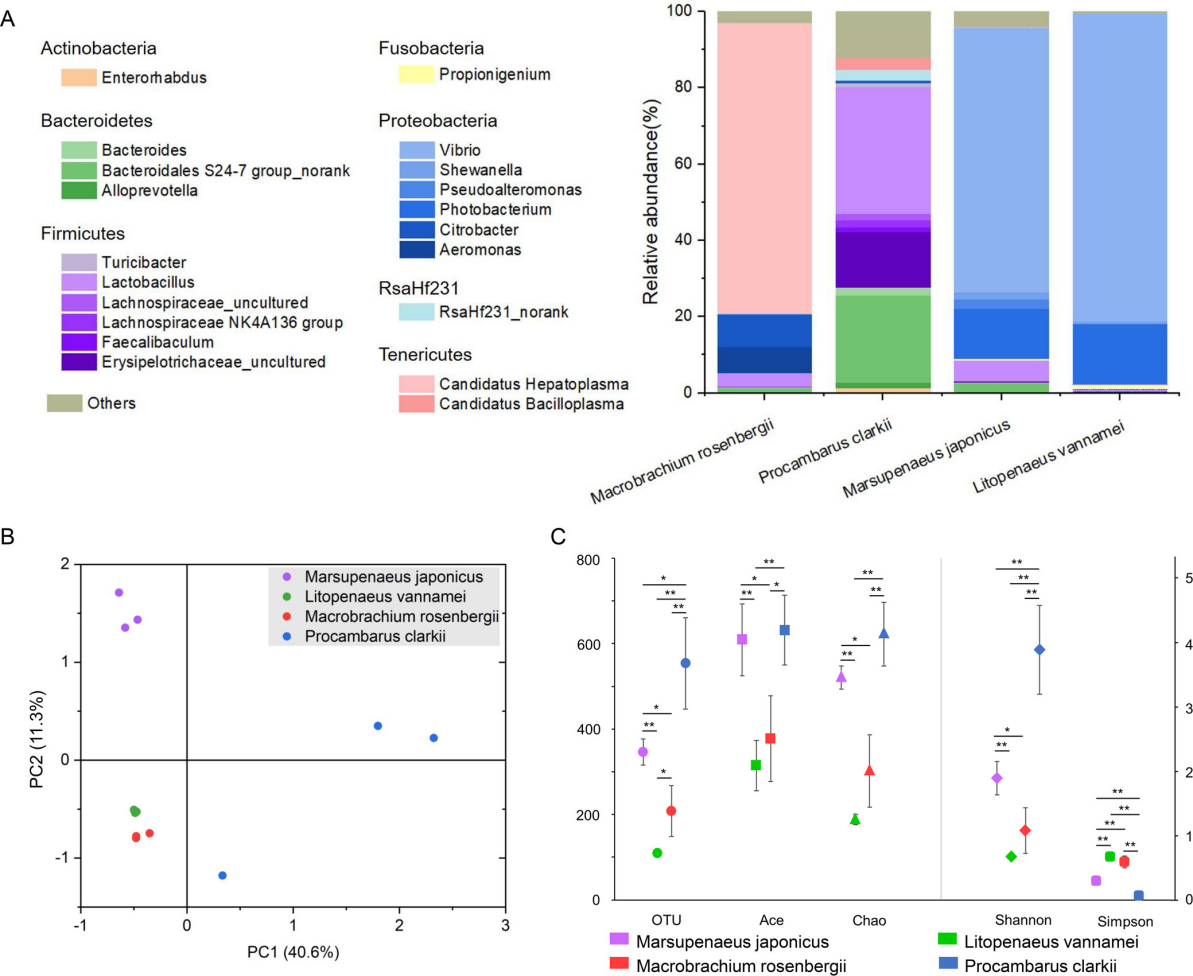
Microbial diversity in the gut among different species of shrimp. (A) The relative abundance of the microbial communities at the genus level found in the guts (*Macrobrachium rosenbergii*, *Procambarus clarkii*, *Marsupenaeus japonicus*, and Litopenaeus vannamei) of different shrimp that are from seawater or freshwater. (B) Principal-coordinate analysis of samples based on the weighted UniFrac method. (C) The α-diversity comparison between different species of shrimp (*n* = 3). Significant differences are indicated by asterisks (*, *P < *0.05; **, *P < *0.01).

In light of exploring the shrimp gut microbiota as a whole, four common shrimp in freshwater and seawater were used in this study: *Macrobrachium rosenbergii*, *Procambarus clarkia*, *Marsupenaeus japonicus*, and Litopenaeus vannamei. Intact intestinal tracts were sampled, and total DNA was extracted followed by 16S rRNA gene amplicon sequencing (*n* = 3). In total, the sequencing of the shrimp gut microbiota yielded 383,028 reads, resulting in 1,135 operational taxonomic units (OTUs; GenBank accession number PRJNA780955). OTUs were classified into 24 phyla and 488 genera. The composition of the microbial communities across the gut revealed significant discrepancies between different species of shrimp (Fig. 1A and B). The dominant bacteria in gut of *Macrobrachium rosenbergii* and *Procambarus clarkii* were *Tenericutes* and *Firmicutes*, respectively. The dominant bacteria of the other two shrimp were both *Proteobacteria* (Fig. 1A). Among the four shrimp species, *Procambarus clarkii* showed significantly higher species richness (paired Wilcoxon *t* test, *P < *0.05; Fig. 1C) and diversity (Shannon index, *P < *0.01), while *Macrobrachium rosenbergii* and Litopenaeus vannamei showed significantly lower richness and diversity (*P < *0.05; Fig. 1C). The diversity and composition of gut microbes may be related to the habitant-intestinal environment of shrimp and suggests that the gut serves as a strong environmental filter, enabling the establishment of distinct microbial communities in the different shrimp.

### A core microbiota persists across different species of shrimp.

**FIG 2:**
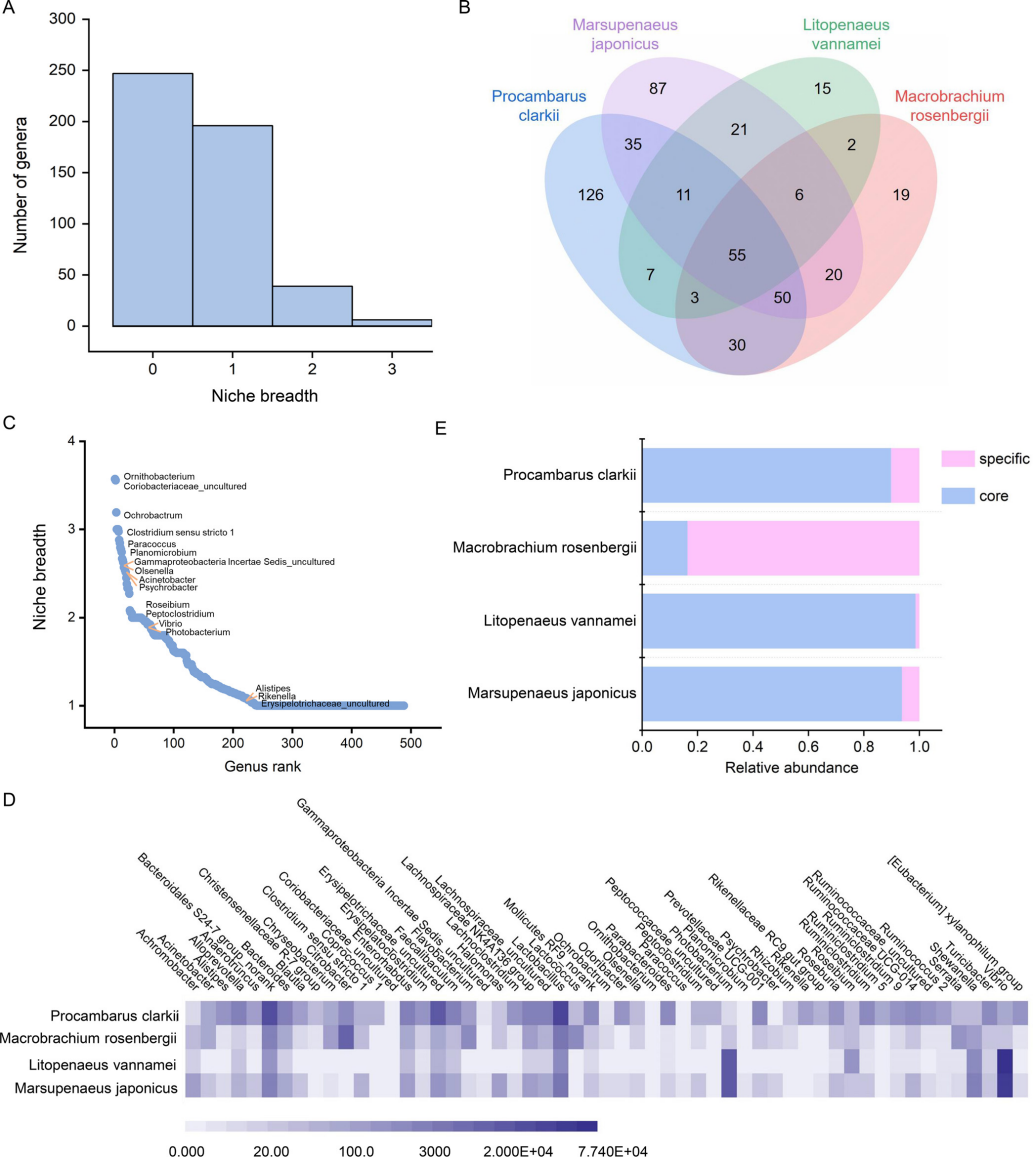
Fifty-five microbes were found as core microbes that persist across the guts of all shrimp species. (A) Niche breadth of the overall microbial communities calculated using Levin’s measure. The larger the niche breadth, the more generalized the microbe. (B) Venn diagram showing the unique and shared bacterial genera in the gut between four shrimp species. At the genus level, 55 microbes that were shared among all habitats. (C) The niche breadth of each microbe, in descending order. The listed microbes are a part of microbes that are found in the guts of all shrimp (core microbe). (D) Heat map showing the abundance of each core microbe across different shrimp guts at the genus level. The shade of color indicates the abundance of microbes. (E) Proportion of core microbes and specific microbes in the guts of different shrimp species.

To identify the core gut microbiota among four species of shrimp, the niche breadth of individual microbes was characterized at the taxonomic level of the genus first, which could reflect the adaptation of species to the environment. In general, the larger the niche breadth of a species, the less specialized it is, that is, the more likely it is to be a generalized species. The niche breadth of each microbe was calculated by using the Levin’s measure, as previously described (8, 25), and the results revealed that only a few of the microbes inhabited a wide range of environments along the different shrimp, and most of them had a narrow range of occupancy across different environments (Fig. 2A), suggesting that microbes with high niche breadth had greater odds of being core microbes. To address this speculation, we used a Venn diagram to analyze the core microbes that exist in every environment and found 55 microbes that fell into this definition (Fig. 2B). Notably, when we examined the correspondence between Venn diagram and niche breadth measurement, we found some inconformity. The niche breadth of some core microbes was lower, such as *Alistipes*, *Rikenella*, *Erysipelotrichaceae_uncultured*, and so on (Fig. 2C), which was due to their uneven distribution in different environments. The abundance of these bacteria could reach up to 9% in *Procambarus clarkii* but less than 1% in the intestines of the other three shrimp species (Fig. 2D). The situation showed that it was the different species of shrimp that shapes various gut microbial communities. Even so, some microbes will not disappear due to this influence, and they were consistently present in the guts of shrimp, although their abundance varied greatly. In this term, 55 such microbes were identified as core microbes in the gut of shrimp (Fig. 2D), and others were identified as specific microbes. Although these microbes contributed almost 90% (except for core microbe in *Macrobrachium rosenbergii* that was due to differences in the dominant microbes) of the total relative abundance, they comprised less than 10% of the overall richness (Fig. 2E). The distribution of these microbes clearly varied in guts of different species.

### Abundance of core microbes in the shrimp gut is changed by WSSV infection.

**FIG 3:**
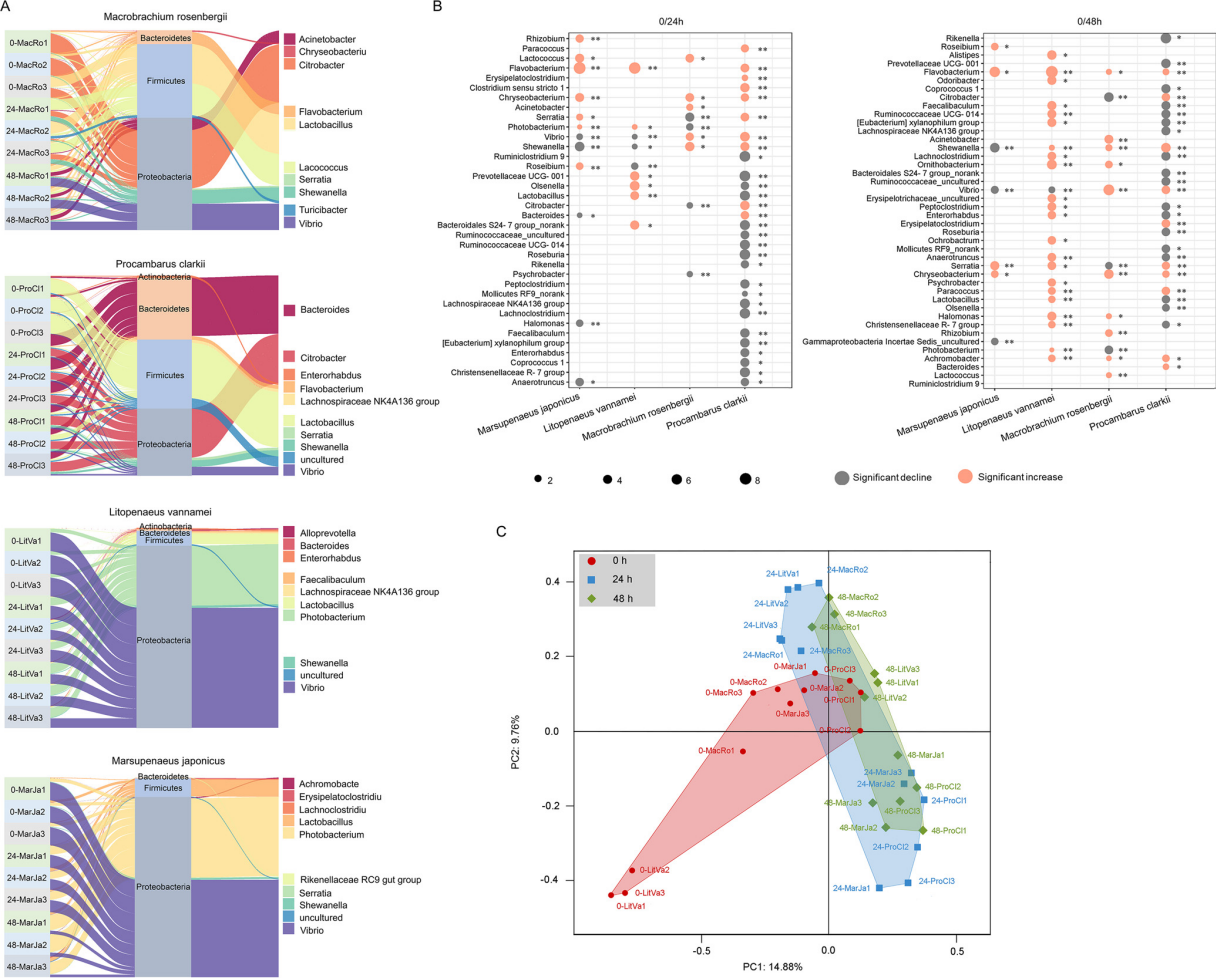
The abundance of core gut microorganisms changed after shrimp were infected with WSSV. (A) Sankey charts of three time points from the core microbial community of the shrimp gut after WSSV infection. The taxonomy level, including phylum and genus, is displayed. The top 10 most abundant genera and the relative changes over time are visible. (B) Balloon plot showing the significant changes of core gut microbial abundance across different shrimp species after WSSV infection. The size of the circles represents the change of bacterial abundance, while the color indicates increase or decline (*n* = 3; *, *P < *0.05 and **, *P < *0.01). (C) Principal-coordinate analysis of samples after WSSV infection.

It is recognized that the gut microbiota is related to pathogen infection in shrimp, but detailed study of the relationship between core microbiota and pathogen infection and their causal roles have not been clearly elucidated. Therefore, WSSV, which is a prevalent viral pathogen and able to cause extremely high mortality in shrimp, was used to explore the issue in this study. Based on the determined core microbiota in the shrimp gut, we analyzed the influence of WSSV infection on core microbial abundance. We found that the abundance of core gut microbes in *Procambarus clarkii* had the greatest disturbance after virus infection, with nearly half of the core microbial abundance significantly changed (*P < *0.01; Fig. 3). Additionally, a Sankey diagram showed some differences in the abundance change of core gut microbes between shrimp from seawater and shrimp from freshwater after WSSV infection (Fig. 3A). The most obvious distinction was in the abundance of *Vibrio* and *Photobacterium*. After virus infection, the abundance of *Vibrio* in the guts of seawater shrimp decreased significantly, and the abundance of *Photobacterium* increased significantly, while the changes in the gut of freshwater shrimp were opposite (Fig. 3A). A similar condition was seen in other microorganisms, such as *Citrobacter*, *Lactobacillus*, *Bacteroides*, and so on. The abundance changes induced by WSSV infection were consistent in some core microorganisms, such as *Achromobacter*, *Chryseobacterium*, and *Flavobacterium* (Fig. 3B). Although the changes were significant, their abundance in the guts of shrimp were relatively low, so the response to virus infection may be small (Fig. 3B). Notably, the taxonomic composition of the microbial community in shrimp after virus infection showed distinct successional trajectories (Fig. 3C), with all microbial communities of the gut developing toward the first and fourth quadrants of the principal-coordinate analysis (PCoA), suggesting that the responses of gut microorganisms among different shrimp to viral infection may eventually follow the same trend.

### Virus infection disrupted the initial metabolism of the shrimp gut.

**FIG 4:**
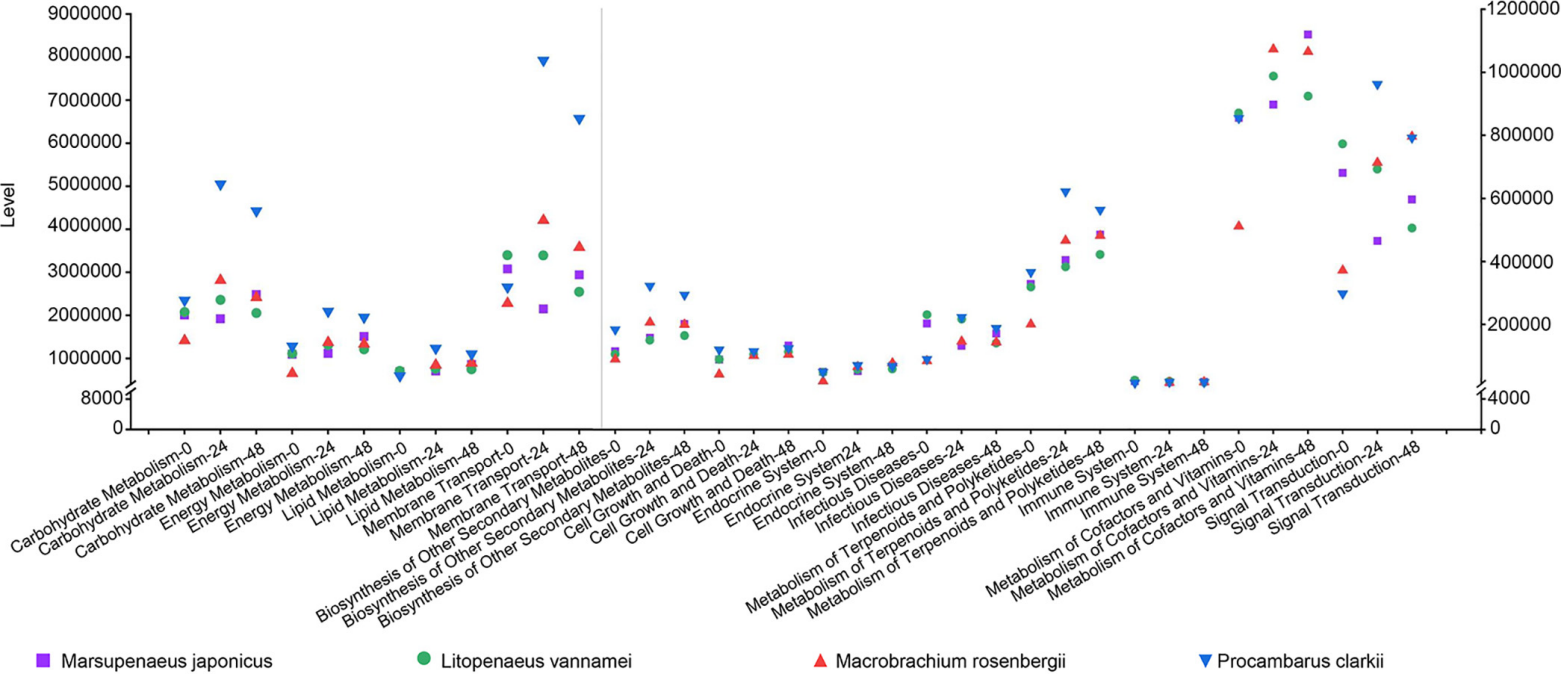
Scatter plot showing the alterations of KEGG metabolic pathways among four species of shrimp after virus infection. Purple, green, red, and blue represent *Marsupenaeus japonicus*, Litopenaeus vannamei, *Macrobrachium rosenbergii*, and *Procambarus clarkia*, respectively.

As reported, the gut microbiota plays a major role in amino acid metabolism, lipid metabolism, protein digestion, and the fermentation of complex carbohydrates into short-chain fatty acids (SCFAs) that are important for the health of organisms (26, 27). Hence, the microbial composition obtained by 16S rRNA gene sequencing was used to predict the Kyoto Encyclopedia of Genes and Genomes (KEGG) metabolic pathways that are involved, and the differences between different samples and groups were analyzed. Based on exploring the proportions of each KEGG metabolic pathway (level 2), we found some discrepancies between infected and uninfected shrimp. These discrepancies had some commonalities in the changes of each species of shrimp after treatment with WSSV (Fig. 4). The proportions of 12 pathways were altered in the guts of the four shrimp after virus infection, including biosynthesis of other secondary metabolites, carbohydrate metabolism, cell growth and death, the endocrine system, energy metabolism, metabolism of terpenoids and polyketides, the immune system, infectious diseases, lipid metabolism, membrane transport, and metabolism of cofactors and vitamins and signal transduction (Fig. 4). Some of these pathways have been previously reported to be related to host metabolism and anti-infection and anti-inflammation processes (20). These results reflect metabolic changes that may be caused by alterations in the microbiome of the shrimp gut after viral infection, indicating that the response of the core microbiota to viral stimulation in different shrimp intestines was consistent.

### Alterations in the gut metabolome by microbiota function in maintaining shrimp immune homeostasis.

**FIG 5:**
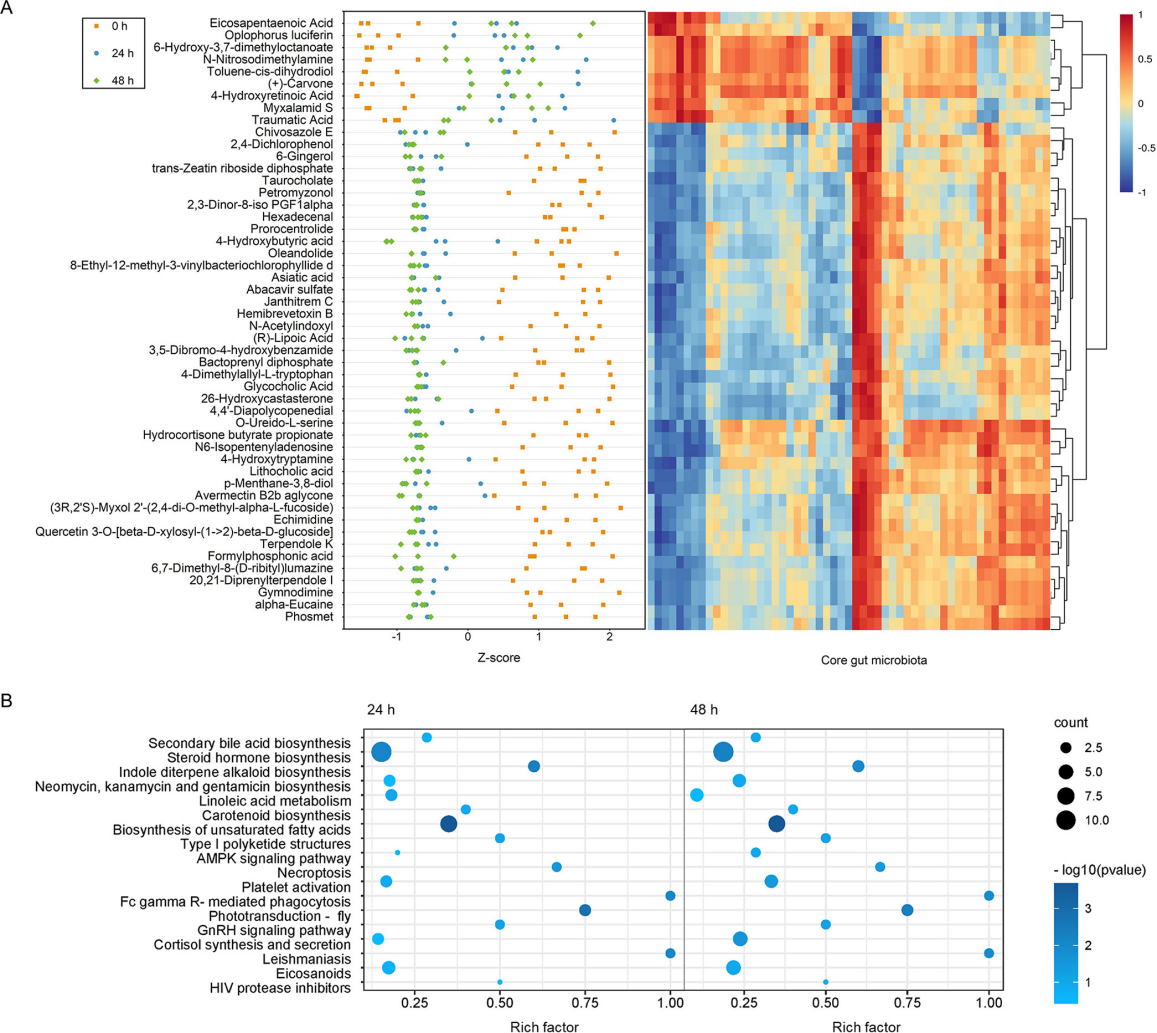
Changes in the intestinal metabolome of *Marsupenaeus japonicus* after WSSV infection. (A) Gut metabolites with significant changes after treatment with WSSV and their correlation with gut core microbiota. The Z-score = (*x* − *μ*)/*σ*, where *x* represents a specific score, *μ* represents the mean value, and *σ* represents the standard deviation. Red boxes indicate positive correlations, while the blue boxes show negative correlations. (B) Enrichment of metabolites in KEGG pathways in response to WSSV infection. The color of the dot represents the *P* value, and the bluer the dot is, the more significant the enrichment is. The size of the dot represents rich number.

By altering the structure of the gut microbiota, infection with WSSV may alter the function of the community. This change in function is reflected in the gut metabolome, which includes both host- and microbial-derived metabolites. To determine which metabolites changed in the guts of shrimp after viral infection and participate in the anti-infection response, the gut metabolome of shrimp was explored using mass spectrometry platforms with an untargeted approach. Shrimp intestinal contents from different time points after infection with WSSV were used to characterize the changes of the gut metabolome, and the top 50 metabolites with maximum fold change and significant changes were selected for display (Fig. 5A). Most metabolites were seen with significant decreases after WSSV infection, some of them varying by more than 50-fold, such as 4-dimethylallyl-l-tryptophan, bactoprenyl diphosphate, gymnodimine, and so on (Fig. 5A). The most significant increase (with 5-fold to 11-fold) was seen in nine metabolites, including toluene-*cis*-dihydrodiol, eicosapentaenoic acid, (+)-carvone, myxalamid S, *N*-nitrosodimethylamine, oplophorus luciferin, traumatic acid, 4-hydroxyretinoic acid, and 6-hydroxy-3,7-dimethyloctanoate, suggesting that they have functional potential in anti-infection of WSSV in the shrimp gut (Fig. 5A). Metabolites that change significantly mostly belonged to 18 KEGG metabolic pathways compared with the initial states, such as secondary bile acid biosynthesis, linoleic acid metabolism, biosynthesis of unsaturated fatty acids, necroptosis, and the AMP-activated protein kinase (AMPK) signaling pathway that are correlated with the immunity and health of organisms, consistent with what the sequencing predicted (Fig. 4 and 5B). Additionally, a correlation analysis conducted with the Spearman algorithm found that the alteration of the gut metabolome was tightly correlated with the changes of the core gut microbiota (Fig. 5A). For example, nine significantly upregulated metabolites after infection strongly positively correlate with half of the core microbes of the gut, while those significantly downregulated metabolites have a strong positive correlation with the rest of the microbes (Fig. 5A). These results showed that despite that the decrease in the large number of metabolites and the enriched KEGG pathway that they belonged to resulted in a reduction in shrimp immunity and health after WSSV infection, the increase of some antiviral metabolites under the influence of gut microbes may assist host adaptation to environmental challenge.

## DISCUSSION

In hosts, multiple factors shape the diversity of the gut microbiome, such as the developmental cycle, dietary differences, intestinal pH, and geographical location of the host (28). The microorganisms that are prevalent among different hosts regardless of these factors are defined as core microbes (8). A functional core microbiota was provided by abundant bacterial taxa. To date, identifications of core gut microbiota typically focus on vertebrates, and information about those of invertebrates is very limited, especially in aquatic invertebrates. Thus, shrimp, an essential invertebrate in global aquatic aquaculture (29) were used to explore the core microbiota in our study. Based on 16S rRNA gene sequencing, the gut microbial composition of four different species of shrimp was determined, and we found that the gut environment of different shrimp is a strong habitat filter for the microbial community, resulting in the high diversity of the gut microbial community among different species of shrimp. By calculating the niche breadth of microorganisms and analyzing their presence or not in different guts, a core gut microbiota containing 55 genera was identified. They were present in the guts of each shrimp species regardless of the environment changes, even though their abundance varies (inhomogeneity) from high to low. Therefore, our study provides a characterization of the core microbiota among different species of shrimp for the first time, and we speculate that they perform a vital role in host response to environmental stimuli.

Recent large-scale studies have provided insights into gut microbial structure and functional potential (30). As reported, the gut microbiota profoundly influences host fitness, and it is able to influence various host physiological processes by regulating multiple processes, including nutrient absorption, immune function, oxidative stress, inflammation, and anabolic balance, when the host is stimulated by the environment (31, 32). The gut microbiota performs its function on some different landscapes in the host, including metabolic, protective, structural, and neurological functions. For instance, some gut microbial species are the main producers of short-chain fatty acids (SCFAs; formate, acetate, propionate, butyrate, valerate, isovalerate, and hexanoate) and function in biodegradation of various undigested organics, bile salt, choline, polyphenols, and so on (33–35). Activities of the gut microbiota have an influential role in modulating the gut-brain axis and in maintaining gut homeostasis in the human body via production, expression, and turnover of these metabolites (36–38). Additionally, activation of goblet cells to secrete mucin is associated with fucose, a product that is cleaved from glycans by gut microbes (39). In shrimp, biofilm formation by gut bacteria was found to be related to regulating homeostasis in invertebrates (40). Shrimp white feces syndrome was also reported to be related to intestinal microbiota dysbiosis (16). Wang’s study had shown that the growth and developmental of shrimp is accompanied by alterations in the gut microbiota, and some pivotal microbes are crucial in the growth of shrimp (24). However, little is known about the response of the gut microbiota to environmental changes, such as viral infection, and its effects on the host. Herein, we found that the core gut microbiota in different species of shrimp adjusts and develops toward the same trend when the host is exposed to virus infection. Such an alteration ultimately leads to downregulation of a large number of metabolites in the gut when the shrimp was infected with viruses, and these metabolites are involved in multiple immune-related pathways. However, the upregulation of nine metabolites and the closely correlated core microbes suggests that they have antiviral function potential and play an important role in host resistance to viral stimulation. This reflects an adaptive adjustment of gut microbes to the environmental challenges of shrimp, which may be manifested as a coadaptation of the host and gut microbes under environmental challenges.

### Conclusions.

In this study, WSSV infection was used as an environmental challenge for four different species of shrimp (*Marsupenaeus japonicus*, Litopenaeus vannamei, *Macrobrachium rosenbergii*, and *Procambarus clarkii*). By using 16S rRNA gene sequencing and liquid chromatography-coupled mass spectrometry (LC-MS), the gut microbiota and metabolome before and after virus infection were identified. Our findings provided the first attempt to compare the gut microbiota among the four different species of shrimp (*Marsupenaeus japonicus*, Litopenaeus vannamei, *Macrobrachium rosenbergii*, and *Procambarus clarkii*), found huge variations, and determined a core gut microbiota composed of 55 microbes. The environmental challenge of WSSV infection led to changes in core microbial structures, but the alteration of core microbiota among different shrimp species followed the same trend and showed immune-related function in the prediction of its metabolic potential. In metabolomic analysis, nine significantly upregulated metabolites that were found after viral infection suggested that they have antiviral potential. Moreover, the tight correlation between them and almost half of the core microbiota demonstrated that they were responsible for maintaining immune homeostasis. These valuable findings greatly enhanced our understanding of the gut microbiota in maintaining host fitness under environmental challenge and provide a new strategy for prevention and treatment of viral infection in shrimp.

## MATERIALS AND METHODS

### Shrimp culture and WSSV infection.

Shrimp were cultured as previously described (18), and three from each group were randomly selected for PCR detection of WSSV with specific primers (5′-TTGGTTTCATGCCCGAGATT-3′ and 5′-CCTTGGTCAGCCCCTTGA-3′) to ensure that the shrimp used for experiments were WSSV free. WSSV-free shrimp were infected with WSSV (10^5^ copies/mL) by injection (100 μL of WSSV inoculum/shrimp) into the lateral area of the fourth abdominal segment. The WSSV-infected shrimp were collected for later experiments at different times after infection.

### Sample collection and DNA extraction.

Three shrimp were randomly selected from each group for aseptically collecting intestines after sterilizing the surface of shrimp with 70% ethanol. The intestine was dissected using sterile instruments, and microbial DNA was isolated from gut samples using the bacterial genome DNA extraction kit (Generay, China) following the manufacturer’s protocols.

### Sequencing and data analysis of microbial 16S rRNA.

Amplicon sequencing covering the V4-V5 regions of bacterial 16S rRNA gene was performed by Mingke Biotechnology Co., Ltd. (Hangzhou, China), using universal bacterial primers 515F (5′-GTGCCAGCMGCCGCGG-3′) and 907R (5′-CCGTCAATTCMTTTRAGTTT-3′). Sequencing was performed using an Illumina PE250 (Illumina, USA), and the barcoded library was constructed using an Illumina TruSeq DNA library kit (Illumina, USA) (41). Sequencing data were uploaded to NCBI (GenBank accession number PRJNA780955).

The paired-end reads were overlapped to assemble the sequences using the Flash program. After removal of low-quality fragments, spacers, primers, and the sequences shorter than 50 bp, the remaining sequences were denoised and screened for chimeric sequences with the pre.cluster command and chimera.uchime command in Mothur. The candidate sequences were classified into operational taxonomic units (OTUs) by 97% sequence similarity using the Usearch program.

### Principal-coordinates analysis.

Beta diversity was evaluated by principal-coordinates analysis (PCoA) plots based on unweighted UniFrac metrics using the vegan of package R (version 3.4.4; https://www.r-project.org/). The potential principal components that affect the difference of sample community composition were found out through dimension reduction based on Euclidean distance and other distances.

### Alpha diversity analysis.

Alpha diversity includes a series of statistical analysis indices to estimate the species abundance and diversity of the environmental community. Community richness was calculated using the following indices: Chao (http://www.mothur.org/wiki/Chao) and Ace (http://www.mothur.org/wiki/Ace). The indices used to calculate community diversity were Shannon (http://www.mothur.org/wiki/Shannon) and Simpson (http://www.mothur.org/wiki/Simpson).

### The calculation of niche breadth.

The niche breadth was calculated as the formula described. The core bacteria inhabit a wide range of environments along the different samples, such as environmental types, and specific bacteria have a narrower range of occupancy across these different environments. *B_i_* represents niche breadth (1 ≤ *B_i_* ≤ *n*), *n* represents the number of habitats, and *i* represents the microbial genus. *P_ij_* is the ratio of genus *i* in the *n*th habitat to the total number of this genus in all habitats (25).

$$\begin{array}{l} B_{i}=1/\sum_{j=1}^{n}{\left(P_{\mathrm{ij}}\right)}^{2}\\ P_{\mathrm{ij}}=n_{\mathrm{ij}}/P_{i+} \end{array}$$

### Functional prediction by PICRUSt2.

The PICRUSt2 (phylogenetic investigation of communities by reconstruction of observed states, v2.1.0-b) pipeline was used to predict functional potentials of the gut microbiota. Functional profiles were predicted using the script picrust2_pipeline.py, generating a table of KEGG orthologs (KOs). KEGG Mapper was used to reconstruct KEGG reference categories (KEGG level 1) and modules (KEGG level 2) according to the KO annotations.

### Metabolome analysis of shrimp intestinal contents based on LC-MS.

Three shrimp were randomly selected from each group, and their intestines were dissected. Intestines (100 mg) were diluted with 1 mL of a mixture of methanol-acetonitrile-water (2:2:1 [vol/vol]), followed by centrifugation at 13,000 rpm for 15 min at 4°C. Subsequently, 100 μL of the supernatant was harvested for liquid chromatography-coupled mass spectrometry (LC-MS) analysis. The metabolic profiles were performed by Mingke Biotechnology Co., Ltd. (Hangzhou, China), on an Agilent 1290 Infinity LC system and Accurate-Mass QTOF/MS-6545 (Agilent Technologies, USA). For chromatographic separation, a C_18_ (2.1 mm × 100 mm) reversed-phase column (Thermo Scientific, USA) preheated at 35°C was used. A prepared sample of 1 μL was injected and maintained at 35°C for analysis. The gradient conditions for elution were 95% acetonitrile for 2 min, 95 to 90% from 2 to 3 min of linear gradient, 90 to 30% from 3 to 9 min, 10% from 10 to 12 min, 10 to 95% from 12 to 12.1 min, and 95% from 12.1 to 14 min. The mobile phase for negative ion mode (ES−) and positive ion mode (ES+) was composed of water with 0.04% formic acid as solvent A and acetonitrile with 0.04% formic acid as solvent B, and the flow rate was at 0.3 mL/min.

### Metabolome data processing.

The original data were converted into *m*/*z* format by ProteoWizard, and peak detection, alignment, and retention time correction were carried out by the XCMS program. The “SVR” method was used to correct the peak positions, and the peaks with a loss rate of >50% in each group were filtered. After correcting the screened peaks, the metabolite identification was obtained by searching the Metlin metabolite database. Statistical analysis was performed by the R program.

### KEGG annotation of differential metabolites.

Different metabolites interact with each other in organisms and form different pathways. The KEGG database was used to annotate the metabolic pathways of the differential metabolites involved. KEGG pathway enrichment was conducted according to the results of differential metabolites. Metabolites with significant alterations between groups were defined as differential metabolites and were obtained at a variable influence on projection (VIP) of >1.5, with a *P* value of <0.05 (*t* test statistics) based on the peak intensities.

### Data availability.

The data we obtained from next-generation sequencing were uploaded to the NCBI database under GenBank accession number PRJNA780955.
